# Student adaptation to college and coping in relation to adjustment during COVID-19: A machine learning approach

**DOI:** 10.1371/journal.pone.0279711

**Published:** 2022-12-30

**Authors:** Yijun Zhao, Yi Ding, Hayet Chekired, Ying Wu

**Affiliations:** 1 Computer and Information Sciences Department, Fordham University, New York, New York, United States of America; 2 Graduate School of Education, Fordham University, New York, New York, United States of America; Federal University of Paraiba, BRAZIL

## Abstract

The COVID-19 pandemic has presented unprecedented challenges for university students, creating uncertainties for their academic careers, social lives, and mental health. Our study utilized a machine learning approach to examine the degree to which students’ college adjustment and coping styles impacted their adjustment to COVID-19 disruptions. More specifically, we developed predictive models to distinguish between well-adjusted and not well-adjusted students in each of five psychological domains: academic adjustment, emotionality adjustment, social support adjustment, general COVID-19 regulations response, and discriminatory impact. The predictive features used for these models are students’ individual characteristics in three psychological domains, i.e., Ways of Coping (WAYS), Adaptation to College (SACQ), and Perceived Stress Scale (PSS), assessed using established commercial and open-access questionnaires. We based our study on a proprietary survey dataset collected from 517 U.S. students during the initial peak of the pandemic. Our models achieved an average of 0.91 AUC score over the five domains. Using the SHAP method, we further identified the most relevant risk factors associated with each classification task. The findings reveal the relationship of students’ general adaptation to college and coping in relation to their adjustment during COVID-19. Our results could help universities identify systemic and individualized strategies to support their students in coping with stress and to facilitate students’ college adjustment in this era of challenges and uncertainties.

## Introduction

The COVID-19 pandemic created numerous unprecedented challenges for university students in particular. For example, thousands of U.S. universities suspended their in-person classes and activities, with some institutions announcing that all classes would be held online for the remainder of the Spring 2020 semester and through the summer session. A large number of university residence halls were closed in response to the suspension of in-person classes, requiring its students to vacate their dorm rooms with only a few days’ notice [1, 2]. Because of the uncertainties imposed by the pandemic [3], students were concerned about the trajectory of their academic careers. Such concerns stemmed from inefficient online learning; suspended fieldwork, internships, and clinical rotations; financial burdens; uncertain living situations; and, for international students, possible changes in visa status [4, 5]. As a result, many students faced heightened levels of psychological distress and changes in behavioral patterns, which called for increased engagement in coping strategies. In terms of coping styles, it is believed that individuals’ coping styles and adjustment processes play important roles in their responses to stress and overall well-being [6], especially during disruptive circumstances such as the COVID-19 pandemic. We were interested in examining how college students’ adjustment processes and coping styles impacted their perceived stress and responses to the COVID-19 pandemic.

The overarching goal of this study is to investigate how participants’ responses to Ways of Coping (WAYS; [6]), the Student Adaptation to College (SACQ; [7]), and the Perceived Stress Scale (PSS; [8]) predict the level of challenges students encountered during COVID-19. In particular, WAYS is an instrument to explore participants’ cognitive and behavioral patterns in managing stressful events. SACQ is an instrument to evaluate students’ adaptation to the university experience. PSS is an instrument to examine participants’ perceived stress levels. For our study, we administered an extensive survey containing questions measuring WAYS, SACQ, PSS, and five COVID adjustment domains (i.e., academic adjustment, emotionality adjustment, social support adjustment, general COVID-19 regulations response, and discriminatory impact) to U.S. students during the initial peak of the pandemic. Many studies focusing on coping and adjustment among college students have used traditional statistical approaches such as correlation and regression analyses. In recent years, social scientists are increasingly interested in utilizing novel machine learning techniques to accurately predict or detect patterns in real-word phenomena [9]. Machine learning offers a wide range of alternative models that might provide substantial improvement in accuracy [10].

In this study, we develop machine learning models to classify well-adjusted and not well-adjusted students in each of the five COVID-19 study domains based on a student’s characteristics in WAYS, SACQ and PSS. We further analyze the most relevant risk factors associated with each classification task. Our findings could help universities to establish systemic and individualized strategies to support their students in coping with stress and adjustments in this era of challenges and uncertainties.

### Theory of ways of coping

A person’s cognitive and behavioral efforts to manage stressful events can be described as ways of coping [11, 12]. Lazarus and Folkman [12] proposed a transactional model of stress and coping to explain individuals’ use of conceptualizations and behavioral responses to manage perceived stressors. The model includes three major categories of coping strategies that individuals generally engage in, namely emotion-focused coping, problem-focused coping, and avoidance-focused coping [12]. Emotion-focused coping focuses on strategies to control emotional responses whereas problem-focused coping involves active efforts to alter the stressful event. Avoidance-focused coping includes strategies to escape from the situation to avoid the stress [6, 13, 14].

To better explore different coping styles and their psychological aftermath, Folkman and Lazarus [6] developed the Ways of Coping Questionnaire (WAYS), which includes a total of eight coping strategies stemming from the three major categories: confrontive coping, distancing, self-controlling, seeking social support, accepting responsibility, escape-avoidance, planful problem solving, and positive reappraisal. Studies have shown that those who engage in proactive problem-focused coping and positive emotion-focused coping generally perceive less stress during a stressful event whereas those who engage in more reactive emotion-focused coping perceive more stress [15].

### Theory of SACQ

It is believed that students generally experience a series of changes and adjustments during their transitions from high school to college [16]. This adjustment process is multifaceted and is closely related to students’ overall university experiences [17]. Areas involved in the adjustment process include emotional well-being, different social expectations, and novel academic requirements [16]. Research has shown that university success is best measured through a combination of students’ cognitive capacity and academic achievement, and those who experience difficulty with university adjustment may choose to drop out of school [16, 18, 19].

To assess how well students were adapting to their novel university experiences, Baker and Siryk [18] developed a quantitative measure, later known as the Student Adaptation to College Questionnaire (SACQ; [7]), and identified four factors closely related to university adjustment: academic adjustment, social adjustment, personal-emotional adjustment, and attachment to the institution. Academic adjustment refers to students’ ability to adhere to the diverse educational demands stemming from university expectations. It is closely related to students’ academic self-efficacy and skills, self-appraisal, motivation to learn, general satisfaction with the academic environment, and educational goals [7, 16]. Social adjustment refers to students’ ability to cope with the interpersonal-societal demands of college life. It is closely related to students’ sense of self-confidence in social situations and ability to cope with stressors, and can be used to predict students’ level of persistence in their university experiences. Personal-emotional adjustment refers to students’ psychological and physical well-being during their university adjustment process and examines students’ coping skills, distress level, and emotional reliance on others. It is related to students’ general school performance, ability to cope with stressors, and overall functioning [7]. Institutional attachment refers to students’ level of commitment to their university and assesses the relationship quality between students and their universities [7]. Studies have shown that students who report higher attachment to and satisfaction with their institutions tend to perceive better social connection, acceptance, and academic competence; possess more coping strategies; and experience fewer negative psychological states [20].

### Theory of perceived stress

Individuals perceive stress when they interpret the presented situational demands as beyond their own capacity to navigate [12]. The level of stress perceived is determined by the individual’s personal conceptualization about the general stressfulness of their life, their ability and confidence to cope with the stress, and their current functioning in a given period of time [8, 12]. In other words, each individual perceives the same stressor, such as the COVID-19 pandemic, differently based on their personal circumstances and beliefs.

## Materials and methods

### Demographic questionnaire

This study utilizes a proprietary survey dataset collected from March to June 2020 during the initial peak of the COVID-19 pandemic. Institutional Review Board (IRB) approval was obtained from Fordham University for data collection and sharing protocols. Eligible participants were at least 18 years of age and enrolled as undergraduate or graduate/professional students at colleges or universities in the United States. All participants completed the SACQ, PSS, WAYS, and the COVID-19 Adjustment questionnaire. The following sections describe the details of these four questionnaires whose data are used in the current study.

In addition, Cronbach’s alpha (α) was used as a reliability measure of internal consistency. It is commonly used to measure how closely related a set of items are as a group in an instrument [21]. Formally, Cronbach’s alpha is defined as:

$$\alpha =\frac{N*\bar{c}}{\bar{v}+\left(N-1\right)*\bar{c}}$$

where *N* is the number of items, $\bar{c}$ is the average inter-item covariance among the items and $\bar{v}$ equals the average variance across all items. In practice, *α* ≥ .70 is considered acceptable for most social science research instruments.

### Student Adaptation to College Questionnaire (SACQ)

The Student Adaptation to College Questionnaire (SACQ; (7]) was administered to evaluate the students’ process of adaptation to the university experience during the first wave of COVID-19. The SACQ is a 67-item self-reported questionnaire with a 9-point Likert scale, ranging from *doesn’t apply to me at all* to *applies very closely to me*, and consists of four subscales: Academic Adjustment (α = 0.88), Social Adjustment (α = 0.91), Personal-emotional Adjustment (α = 0.87), and Institutional Attachment (α = 0.90). Participants’ responses were summed and then were converted into *T*-scores, with higher scores indicating higher levels of adjustment [7, 18].

### Perceived Stress Scale (PSS)

The Perceived Stress Scale (PSS; [22]) was administered to examine participants’ perceived stress levels during the initial peak of the COVID-19 pandemic. The PSS is a 10-item self-report questionnaire with a 5-point Likert-scale ranging from *never* to *very often* ([8]; α = 0.87). According to Cohen et al. [8], higher summed scores indicate higher levels of stress and lower scores indicate lower levels of stress. The questions on the PSS are context free (i.e., questions were not worded to fit specific circumstances and they were generic questions), enabling its usage with any subpopulation group. By focusing on the participants’ current thoughts and feelings, the PSS is intended to explore the participants’ perceptions of the degree of unpredictability, uncontrollability, and overwhelmingness of their life experiences during the past 30 days. In this study, two scores were derived upon analysis, with the total score including all 10 items and the short score including four selected items. The total score was summed with appropriate items scored as indicated by Cohen [22]. The short score was derived from the four items that showed the highest correlation with the full-scale items examined by Cohen et al. [8], and it generates general inquiries about the respondents’ experiences of relative current levels of stress.

### Ways of Coping Questionnaire (WAYS)

The Ways of Coping Questionnaire (WAYS; [6]) was used to explore participants’ coping strategies and processes during the initial peak of the COVID-19 pandemic. WAYS is a 66-item self-reported questionnaire with a 4-point Likert scale ranging from *does not apply or not used* to *used a great deal* ([23]; α = 0.78). Of note, the WAYS was developed with the specific focus on participants’ actual and/or potential actions in response to a stressful situation rather than their thoughts and feelings about the situation [23–26]. Eight subscales were generated based on factor analyses: Confrontive, Distancing, Seeking Social Support, Accepting Responsibility, Positive Reappraisal, Planful Problem Solving, Escape Avoidance, and Self-Controlling [11]. In the current study, participants’ responses were accumulated to obtain a score for each subscale, with higher scores suggesting their inclination to use the coping behaviors defined by that subscale when encountering COVID-19-related stressors [6].

### COVID-19 adjustment questionnaire

A self-report questionnaire with a 5-point Likert-scale ranging from *strongly disagree* to *strongly agree* was created for the original larger-scale study to measure the effects of COVID-19 on participants and their adjustments [27]. This questionnaire was adapted from an unpublished instrument created to measure university students’ experiences and mental health during the initial COVID-19 outbreak in China [27]. Factor analyses on the original questionnaire yielded five subdomains: Academic Adjustment, Emotionality Adjustment, Social Support, General COVID-19 Regulations Response, and Discriminatory Impact Related to COVID-19. The academic adjustment subscale (7-item, α = .85) measured the degree to which participants’ felt prepared and motivated to complete academic work and ability to adjust to remote education as a result of the COVID-19 pandemic (e.g., “I have a virtual-learning supportive atmosphere at home (e.g., computer, wifi, quiet space)). The emotionality subscale (4-items, α = .71) measured participants’ ability to deal with emotional thoughts and behaviors towards COVID-19 related stimuli and experiences (e.g., “I feel like the Coronavirus is far from me.”). The social support subscale (4-items, α = .69) measured participants’ level of satisfaction with received support during the COVID-19 pandemic (e.g. “I feel supported by my professors and university”). The general regulation reaction subscale (3-items, α = .61) measured participants’ agreement with regulations and restrictions imposed due to the COVID-19 pandemic (e.g., “I feel relieved that schools are closed and classes have moved online.” The discriminatory impact adjustment subscale (3-items, α = .78) measured participants’ acknowledgement and impact of racism as related to COVID-19 (e.g., “I am aware of Asians’ experience with discrimination due to the coronavirus.”) Participants were believed to be adjusting more positively during the COVID-19 pandemic if they reported a high score on these subdomains.

### Machine learning methods

To build our machine learning models, we first labeled each participant as well-adjusted (class 1) or not well-adjusted (class 0) in each of the five COVID-19 adjustment domains. They serve as the output of our predictive models. To accomplish this, we identified the set of questions *Q* in the survey pertinent to each domain and computed the average score of answers to these questions. A participant was labeled as a class 1 instance if their total score for *Q* was above the average. Otherwise, the participant was labeled as a class 0 instance. For example, the academic adjustment domain consisted of seven questions, each with a Likert scale from 1 to 5. Thus, the average score for this domain was 7 x 3 = 21, where 7 was the number of questions and 3 was the middle score of each question. Class 1 instances for this domain were those participants whose total score for the seven questions was above 21. Table 1 presents the distribution of participants for each of our classification tasks.

**Table 1:** Distribution of participants across classes in each study domain.

COVID-19 Adjustment Domain	# well-adjusted (class 1)	# not well-adjusted (class 0)
Academic	139	378
Emotionality	99	418
Social Support	482	35
COVID-19 Regulations Response	495	22
Discriminatory Impact	47	470

The input features of our classification models are the student’s scores in each subscale of the three non-COVID (i.e., WAYS, SACQ, and PSS) domains. Consequently, the total number of features is 14, with 8, 5, and 2 from WAYS, SACQ, and PSS, respectively. Table 2 shows the list of features employed by this study and their brief descriptions.

**Table 2:** Input features for the classification models.

Index	Feature	Description
1	SACQ_A	Student’s level of affection and connection with their university.
2	SACQ_AA	Student’s adaptation to the educational demands of their university.
3	SACQ_PEA	Student’s psychological and physical well-being in their university experiences.
4	SACQ_SA	Student’s interpersonal and societal demands in their university experiences.
5	PSS_Total	Student’s perceived level of stress in the past 30 days.
6	PSS_Short	An abbreviated measure of student’s perceived level of stress in the past 30 days.
7	WAYS_AR	A coping strategy that uses efforts to reclaim self-worth by engaging in positive cognitive and behavioral changes.
8	WAYS_CC	A coping strategy that uses efforts that are considered aggressive and risky.
9	WAYS_D	A coping strategy that involves actions of removing oneself from a stressful situation.
10	WAYS_EA	A coping strategy that involves wishful thinking and pessimistic behaviors to avoid the stressful situation.
11	WAYS_PPS	A coping strategy that utilizes conscious effort that targets the problem directly to change the stressful situation.
12	WAYS_PR	A coping strategy that involves efforts in focusing on self-growth and creating positive meanings from the stressful situation.
13	WAYS_SC	A coping strategy that involves efforts in regulating one’s own feelings and actions.
14	WAYS_SSS	A coping strategy that involves attempts to reach out to social networks for consultation and validation.

We employed seven established machine learning methods and compared their efficacy for our predictive tasks. Of these, logistic regression [28], support vector machine (SVM; [29]), decision tree [30], random forest [31], neural network [32], and AdaBoost [33] are standalone algorithms. We leveraged their implementations from Python’s *scikit-learn* package [34]. Our last model was a majority-voting ensemble learner [35] based on the aforementioned six models.

All models were trained using a 5-fold (outer) cross-validation. Therein, we divided the training data into five disjoint partitions (i.e., folds) and trained/evaluated each classifier five times with different training and test data. Specifically, at each iteration *i* (*i* = 1, 2, … 5), fold *i* was designated as the test data and the remaining nine folds were designated as the training data. Hyperparameters were selected using a nested 5-fold (inner) cross-validation on the training data. We reported the average performance of the 5 test folds.

### Addressing imbalanced data

A particular challenge in building our classification models was the severe data imbalance in the two classes. For example, Table 2 shows that the ratio between the instances from the two classes was 495/22 = 22.5 for the COVID-19 Regulations Response domain. Standard machine learning algorithms implicitly assume an equal representation of training data for each class. Applying the models directly to severely imbalanced data would lead to unsatisfactory performance on the minority class. We addressed this issue using the oversampling technique. Specifically, each minority sample was duplicated (*r* -1) times to match the total number of majority instances, where *r* was the data size ratio in the two classes. While there are a few other methods, such as SMOTE [36] and bagging [37], to address imbalanced data in building machine learning models, our experiments found that the differences were marginal, and the results in Table 2 confirmed that the oversampling technique was sufficient.

### Evaluation metrics

We evaluated the performance of our classification models using six metrics defined as follows:

- *Overall accuracy*: the fraction of correctly classified instances in the test data.
- *Recall*: the fraction of correctly classified instances among all well-adjusted instances.
- *Specificity*: the fraction of correctly classified instances among all not well-adjusted instances.
- *Precision*: the fraction of correctly classified instances among all positive predictions.
- *F1 score*: harmonic mean of recall and specificity.
- *AUC score (of the ROC curve)*: A ROC curve displays the trade-off between the true positive rate (TPR, or sensitivity) and the true negative rate (TNR, or specificity) of a classification model at different threshold settings. AUC reveals the capability of a model to separate the positive and negative classes; that is, the higher the AUC score, the more effective a model is at performing the classification.

### Model interpretation with SHAP plots

SHAP (SHapley Additive exPlanation) is a unified framework to interpret the predictions of machine learning models [38]. SHAP is based on the classic concept of Shapley value [39], which is used in game theory to fairly distribute gains and costs among a group of collaborating players in achieving a specific goal. Because some players contribute more to the coalition than others and players have different levels of leverage or efficiency, it is important to investigate how essential each participant is to the final outcome. Shapley value quantifies the contribution that each player brings to the game using well-studied mathematically theory and properties (the formal derivation of the calculation is beyond the scope of this paper).

**Fig 1:**
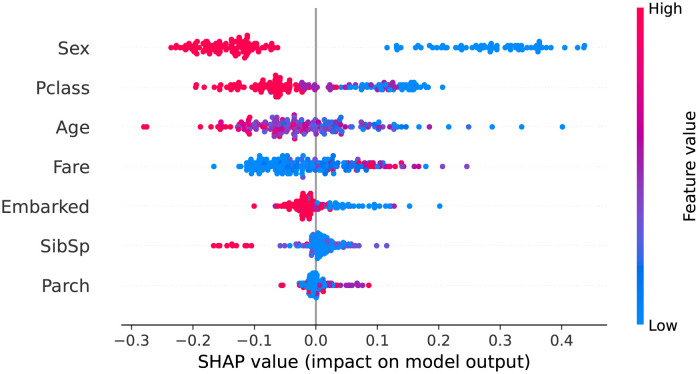
SHAP plot for titanic survival analysis using the random forest model.

Within the machine learning context, a model can be viewed as a coalition game in which the prediction is the goal and the predictive features serve as the players. We are interested in understanding the effect of each feature on the outcome variable. SHAP plot provides an effective method to visualize the feature contributions to the game outcome, which are the model predictions. For instance, Fig 1 presents the beeswarm SHAP plot for the random forest applied to the Titanic dataset [40]. The task is to predict a passenger’s survival using 12 characteristic features (Sex, Pclass, Age, etc.). The predictive features are plotted in their order of relative importance along the y-axis. In addition, each row illustrates a feature’s contribution to the predictive outcomes, with each dot representing an individual instance in the dataset. The magnitude of feature values is color-coded from blue (low) to red (high). The points are distributed horizontally along the x-axis according to their SHAP values. In places where there is a high density of SHAP values, the points are stacked vertically. Examining the color distribution horizontally along the x-axis for each variable provides insights into the general relationship between a variable’s raw values and its directional impact to the outcome. For instance, Fig 1 shows that a passenger’s sex (encoded as male = 0 and female = 1) is the top feature in predicting a passenger’s chances of survival. Lower values (i.e., female) are concentrated on the right side of the y-axis, indicating a high chance of survival. The next two predictive features are Pclass (encoded as first-, second-, and third-class cabins) and age. In particular, low values of Pclass (i.e., high socioeconomic status) are associated with a high chance of survival, and the same is true for low age values. Thus, we can infer that women, children, and passengers in the first-class cabins had a greater probability of survival than the others.

In our study, we employed the SHAP approach to study the relative importance and directional impact of those predictive features utilized in our classification tasks.

## Results

This section presents the results of seven machine learning models on classifying well-adjusted and not well-adjusted students in each of the five COVID-19 study domains based on their characteristics in WAYS, SACQ and PSS. The performance for each classification task was evaluated using the six metrics described above. The analysis focuses exclusively on the performance on the test data. Of these seven models, random forest displayed the highest AUC scores, although the advantage was marginal in some cases. All random forest models used 1000 estimators. The depth of the trees ranged from 14 to 30, and the minimum leaf samples ranged from 10 to 20. These hyperparameters were selected using the method outlined in the “Machine Learning Methods” section. Remaining unspecified hyperparameters assumed the default values in Python’s scikit-learn library—the software we used to build our models. We present the risk factors in each task using the SHAP approach described above.

### Classification task performance

#### Academic adjustment

The results in Table 3 show that random forest achieved the highest overall accuracy (86%) and AUC score (0.86) in predicting students’ academic adjustment. When the results were broken down into performance on the well-adjusted class and the not well-adjusted class, random forest achieved results of 90% (recall) and 82% (specificity), respectively. Random forest also led the other models in precision and F1 scores of 0.84 and 0.87, respectively.

**Table 3:** Performance of seven machine learning models for each classification task.

Model	Overall Accuracy	Recall	Specificity	Precision	F1	AUC
**Academic Adjustment**						
Logistic Regression	0.74	0.75	0.73	0.73	0.74	0.74
SVM (RBF kernel)	0.80	0.85	0.75	0.78	0.81	0.80
Decision Tree	0.80	0.88	0.73	0.77	0.81	0.80
Random Forest	**0.86**	**0.90**	**0.82**	**0.84**	**0.87**	**0.86**
Neural Network	0.81	0.88	0.75	0.78	0.83	0.82
AdaBoost	0.84	0.91	0.78	0.81	0.85	0.84
Ensemble	0.84	0.87	0.82	0.83	0.85	0.84
**Emotionality Adjustment**						
Logistic Regression	0.77	0.78	0.76	0.77	0.78	0.77
SVM (RBF kernel)	0.87	0.94	0.80	0.83	0.88	0.87
Decision Tree	0.86	0.90	0.81	0.83	0.87	0.86
Random Forest	**0.89**	**0.94**	**0.85**	**0.86**	**0.90**	**0.89**
Neural Network	0.87	0.93	0.81	0.83	0.87	0.87
AdaBoost	0.86	0.91	0.81	0.83	0.87	0.86
Ensemble	0.88	0.91	0.84	0.85	0.88	0.87
**Social Support Adjustment**						
Logistic Regression	0.74	0.71	0.78	0.76	0.73	0.74
SVM (RBF kernel)	0.92	0.87	0.98	0.98	0.92	0.92
Decision Tree	0.87	0.84	0.91	0.90	0.87	0.87
Random Forest	**0.93**	**0.87**	**0.99**	**0.99**	**0.93**	**0.94**
Neural Network	0.87	0.81	0.93	0.92	0.86	0.87
AdaBoost	0.85	0.78	0.93	0.92	0.84	0.85
Ensemble	0.85	0.76	0.95	0.94	0.84	0.85
**Regulations Response**						
Logistic Regression	0.72	0.72	0.72	0.72	0.72	0.72
SVM (RBF kernel)	0.90	0.84	0.97	0.96	0.89	0.90
Decision Tree	0.91	0.86	0.97	0.97	0.90	0.91
Random Forest	**0.92**	**0.87**	**0.98**	**0.97**	**0.92**	**0.92**
Neural Network	0.90	0.83	0.98	0.97	0.90	0.90
AdaBoost	0.89	0.84	0.94	0.94	0.89	0.89
Ensemble	0.88	0.81	0.97	0.95	0.88	0.89
**Discriminatory Impact Adjustment**						
Logistic Regression	0.69	0.73	0.66	0.69	0.71	0.69
SVM (RBF kernel)	0.91	0.97	0.84	0.86	0.92	0.91
Decision Tree	0.90	0.93	0.86	0.87	0.90	0.90
Random Forest	**0.93**	**0.96**	**0.91**	**0.92**	**0.93**	**0.93**
Neural Network	0.92	0.98	0.86	0.88	0.93	0.92
AdaBoost	0.92	0.97	0.87	0.88	0.92	0.92
Ensemble	0.92	0.98	0.86	0.88	0.93	0.92

#### Emotionality adjustment

Table 3 shows that random forest achieved the highest overall predictive accuracy (89%) for predicting students’ emotionality adjustment. The model had a 94% accuracy in predicting the well-adjusted class and 85% accuracy for the not well-adjusted class. Random forest also led other models in precision (86%), F1 (90%), and AUC (0.89) scores.

#### Social support adjustment

The results in Table 3 show that random forest led other models in predicting social support adjustment across all six evaluation metrics. More specifically, the model achieved a 0.93 overall accuracy with 0.87 and 0.99 for the well-adjusted and not well-adjusted classes, respectively. The precision, F1, and AUC scores were 0.99, 0.93, and 0.94, respectively.

#### General COVID-19 regulations response

The results in Table 3 show that random forest delivered the best predictive accuracy value of 92% compared to the other models. When this outcome was dissected into performance in the well-adjusted class and the not well-adjusted class, random forest achieved results of 87% and 98%, respectively. Random forest also led the other models in the remaining evaluation metrics with 0.97, 0.92, and 0.92 for precision, F1, and AUC scores, respectively.

#### Discriminatory impact related to COVID-19

Table 3 shows that random forest had the best overall accuracy performance of 93%. The performances for the well-adjusted and not well-adjusted classes were 96% and 91%, respectively. The model also exhibited the highest precision (0.92), F1 (0.93), and AUC (0.93) scores.

### Analysis of predictive features

An additional intention of this research was to identify the most relevant risk factors associated with each aspect of college adjustment, especially during the COVID-19 pandemic. The findings will help university administrators focus on the most effective measures to influence students’ college adjustment during the ongoing COVID-19 pandemic challenges and support the students accordingly. Table 3 represents the top-10 predictors for the five classification tasks using the best performing model (i.e., RF). The bold features in Table 3 are the variables with the most substantial impact whose SHAP values were above the average magnitude.

**Fig 2:**
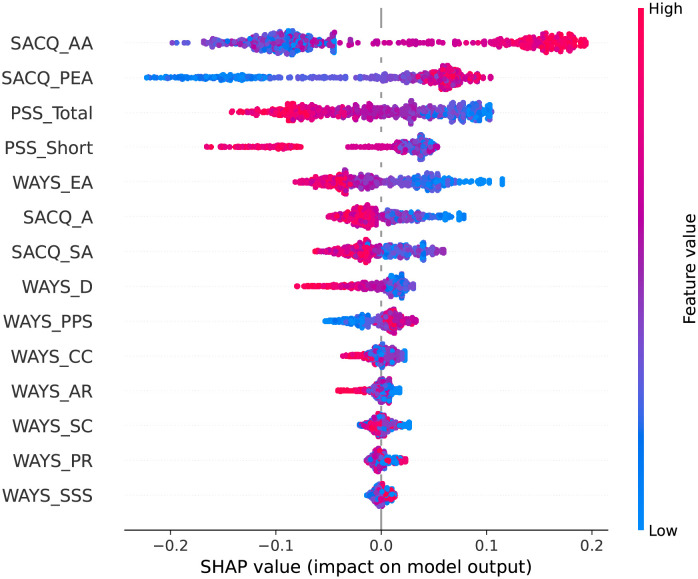
SHAP plot for academic adjustment using the random forest model.

**Fig 3:**
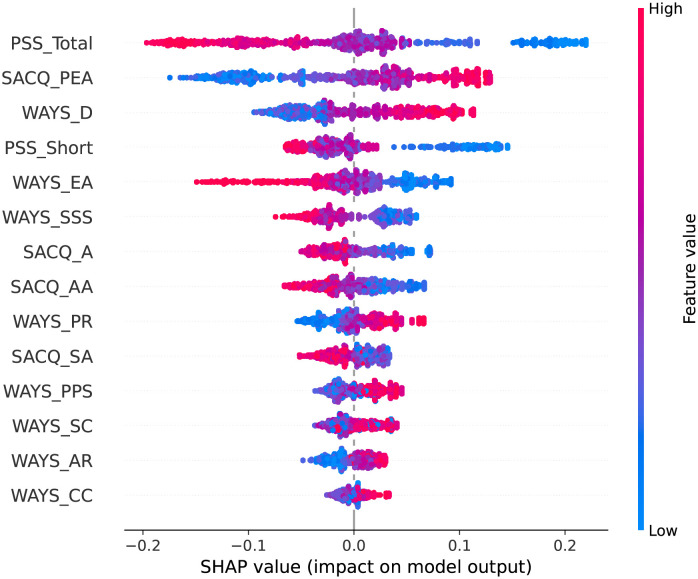
SHAP plot for emotionality adjustment using the random forest model.

**Fig 4:**
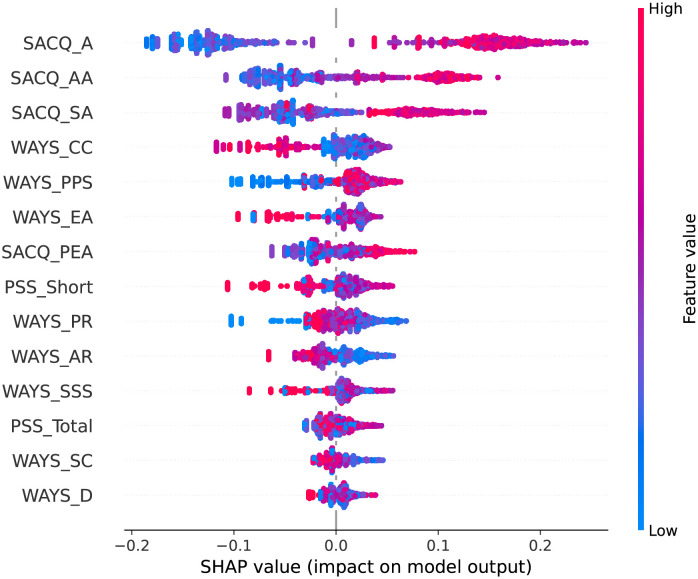
SHAP plot for social support adjustment using the random forest model.

**Fig 5:**
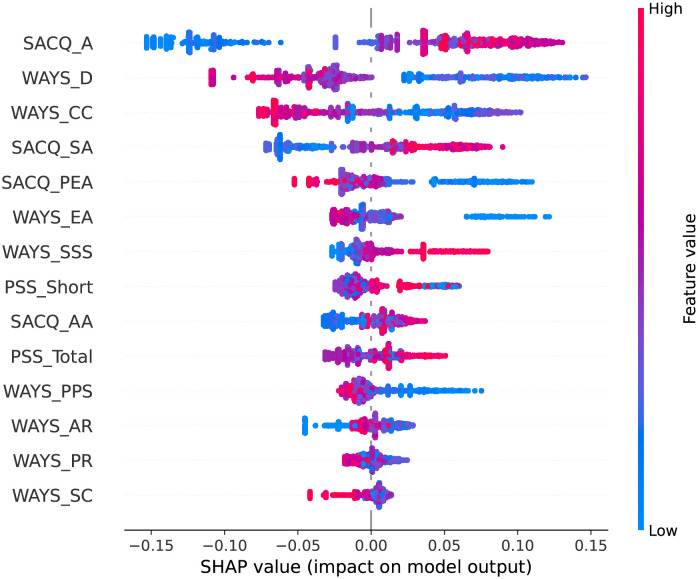
SHAP plot for covid-19 regulations response using the random forest model.

**Fig 6:**
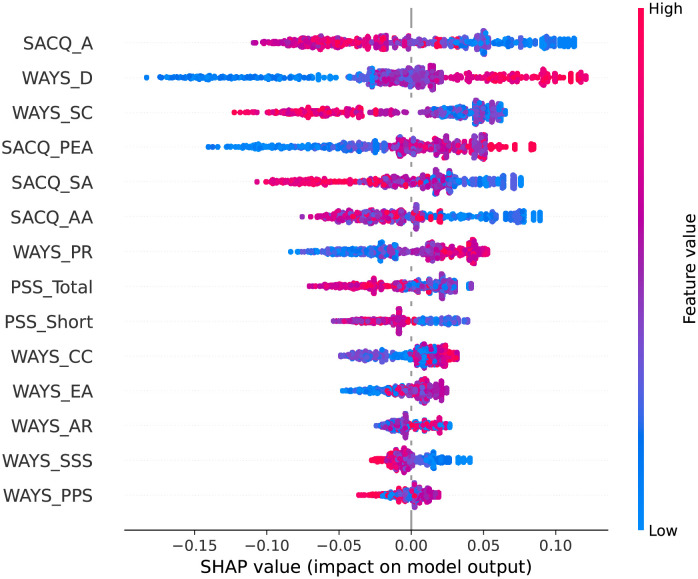
SHAP plot for discriminatory impact using the random forest model.

We present the SHAP plots in Figs 2–6 for the top 10 predictive features of each model in Table 4 to visualize the directional impact of the predictors on the dependent variable. We were particularly interested in distinguishing the positive and negative predictors among the highly ranked (i.e., bold) features for each classification task.

**Table 4:** Top 10 predictive features for each classification task based on random forest model.

Academic	Emotionality	Social Support	Regulations Response	Discriminatory Impact
**SACQ_AA**	**PSS_Total**	**SACQ_A**	**SACQ_A**	**SACQ_A**
**SACQ_PEA**	**SACQ_PEA**	**SACQ_AA**	**WAYS_D**	**WAYS_D**
**PSS_Total**	**WAYS_D**	**SACQ_SA**	**WAYS_CC**	**WAYS_SC**
**PSS_Short**	**PSS_Short**	**WAYS_CC**	**SACQ_SA**	**SACQ_PEA**
**WAYS_EA**	**WAYS_EA**	**WAYS_PPS**	**SACQ_PEA**	**SACQ_SA**
SACQ_A	WAYS_SSS	**WAYS_EA**	WAYS_EA	**SACQ_AA**
SACQ_SA	SACQ_A	SACQ_PEA	WAYS_SSS	WAYS_PR
WAYS_D	SACQ_AA	PSS_Short	PSS_Short	PSS_Total
WAYS_PPS	WAYS_PR	WAYS_PR	SACQ_AA	PSS_Short
WAYS_CC	WAYS_SA	WAYS_AR	PSS_Total	WAYS_CC
WAYS_AR	SACQ_PPS	WAYS_SSS	WAYS_PPS	WAYS_EA
WAYS_SC	WAYS_SC	PSS_Total	WAYS_AR	WAYS_AR
WAYS_PR	WAYS_AR	WAYS_SC	WAYS_PR	WAYS_SSS
WAYS_SSS	WAYS_CC	WAYS_D	WAYS_SC	WAYS_PPS

#### Academic adjustment

For academic adjustment, Table 4 indicates that the random forest model proposes academic adjustment (SACQ_AA), personal-emotional adjustment (SACQ_PEA), both perceived stress features (PSS_Total and PSS_Short), and avoidance coping (WAYS_EA) as the most important predictors in students’ academic adjustment during the COVID-19 pandemic.

Analysis of the SHAP plot (Fig 2) helps to identify the direction of the impact of the features on academic adjustment. The findings show that academic adjustment (SACQ_AA) and personal-emotional adjustment (SACQ_PEA) are positive predictors of college adjustment, meaning that high academic performance and positive emotions lead to positive adjustment to academic changes. However, high levels of stress (PSS_Total and PSS_Short), and avoidance coping (WAYS_EA) lead to difficulties adjusting to college life during the COVID-19 pandemic.

#### Emotionality adjustment

As shown in Table 4, the random forest model suggests that perceived stress variables (PSS_Total and PSS_Short), personal-emotional adjustment (SACQ_PEA), distancing coping (WAYS_D), and escape avoidance (WAYS_EA) are the features with the highest influence on students’ emotionality adjustment prediction during the COVID-19 pandemic.

The SHAP plot (Fig 3) indicates that both perceived stress features (PSS_Total and PSS_short) have a negative impact on emotionality adjustment. Personal-emotional adjustment (SACQ_PEA) and distancing (WAYS_D) are positive predictors meaning that students who are more adjusted in their personal psychological and physical well-being and use distancing coping strategies are likely to experience fewer negative emotions during the COVID-19 pandemic. The plot also shows that escape avoidance (WAYS_EA) has a negative influence, meaning students who use avoidance coping are more likely to experience negative emotions.

#### Social support adjustment

As shown in Table 4, attachment (SACQ_A), academic adjustment (SACQ_AA), social adjustment (SACQ_SA), confrontive coping (WAYS_CC), planful problem solving (WAYS_PPS) and escape avoidance (WAYS_EA) are the strongest predictors based on the random forest model, suggesting that these predictors are the more reliable features in predicting students’ social support adjustment during the COVID-19 pandemic.

The SHAP plot (Fig 4) shows that high levels of attachment (SACQ_A), academic performance (SACQ_AA), social integration (SACQ_SA), and planful problem solving (WAYS_PPS) lead to positive social support adjustment, which means students who are attached to the institution, have satisfactory academic performance, are actively social, and approach problems in a systematic way are likely to perceive more social support. The plot also shows that confrontive coping (WAYS_CC) and escape avoidance (WAYS_EA) are negative predictors.

#### General COVID-19 regulations response

As shown in Table 4, the RF model suggests that institutional attachment (SACQ_A), distancing coping (WAYS_D), confrontive coping (WAYS_CC), social adjustment (SACQ_SA), and personal-emotional adjustment (SACQ_PEA) are the most relevant features in students’ adjustment and response to general COVID-19 regulations during the pandemic.

The SHAP plot (Fig 5) shows that high levels of attachment (SACQ_A) and social adjustment (SACQ_SA) lead to positive adjustment to regulations and restrictions imposed by public health officials while high scores in distancing (WAYS_D), confrontive coping (WAYS_CC), and personal-emotional adjustment (SACQ_PEA) lead to negative adjustment.

#### Discriminatory impact related to COVID-19

As shown in Table 4, the RF model suggests that institutional attachment (SACQ_A), distancing coping strategies (WAYS_D), self-control (WAYS_SC), personal-emotional adjustment (SACQ_PEA), social adjustment (SACQ_SA), and academic adjustment (SACQ_AA) are the most influential factors in students’ adjustment to discriminatory impact related to COVID-19.

The SHAP plot (Fig 6) shows that high levels of institutional attachment (SACQ_A), and social adjustment (SACQ_SA) lead to students experiencing more discrimination impact. Said differently, students who are attached to their institution and actively social are more likely to experience discrimination related to COVID-19. The plot also shows that the same is true for academic adjustment (SACQ_AA) and self-control (WAYS_SC). On the other hand, students who use distancing coping strategies (WAYS_D) and positive emotions (SACQ_PEA) are less likely to experience discrimination.

## Discussion

### Machine learning

Results in Table 3 show that the logistic regression model consistently underperformed other approaches across all five classification tasks. One explanation is that the target variables in our study were not linearly dependent on the explanatory variables. While logistic regression does not require a direct linear relationship between the dependent and independent variables, it still needs independent variables to be linearly related to the log-odds of the outcome. Our findings suggest that non-linear models are more desirable in predicting students’ responses to COVID-19 challenges based on their overall adaptation to college and characteristic coping methods.

Among the non-linear models, the random forest model demonstrated modest advantages over the other models. However, since it is impossible to conduct an exhaustive hyperparameter search, it is reasonable to expect that these models were equally effective for our predictive tasks.

### Academic adjustment

Academic adjustment reflects students’ ability to cope with the various educational demands of the university experience, which correlates with students’ setting of academic goals, self-appraisal, and feelings of control over the outcome of their academic efforts. Academic adjustment consists of factors such as motivation, application, performance, and the academic environment and includes academic goals, academic self-efficacy, and academic-related skills [7, 41]. Analysis of students’ academic adjustment considers students’ attitudes towards their academic goals and their required academic demands, their sense of educational purpose, and their level of satisfaction with their academic environment. How well students’ motivation translates into academic effort and how successful students are in applying their knowledge and skills to their academic work and meeting academic requirements are also important in overall academic adjustment [7]. It is not surprising that academic adjustment is a positive predictor of adaptation to college. The findings underscore the importance of examining college students’ overall academic adjustment, especially when they encounter a stressful public health emergency like the COVID-19 pandemic with its disruptive effects on their university environment.

### Emotionality adjustment

Personal-emotional adjustment consists of students’ psychological and physical well-being and is associated with psychosocial coping skills and resources, degree of experienced psychological distress, and emotional reliance on others [7]. University students display higher rates of depression, anxiety, eating disorders, and other forms of psychological distress than the rest of the population [5, 42]. Psychological and physical well-being impact school performance, overall functioning, and ability to cope with demands and stressors [16, 42]. Experiences related to personal-emotional adjustment with regard to the university experience may have been beneficial in managing perceived stress related to the COVID-19 pandemic. Our findings indicate that personal-emotional adjustment has a considerable influence on COVID-19- related emotionality adjustment prediction.

### Social support adjustment

In terms of social support adjustment, the results indicate that the more attachment students felt towards their university and the better adjusted the students were to their college academics and social lives, the more likely students were to perceive better social support during the COVID-19 pandemic. This result is supported by a vast amount of past literature. For example, Ames et al. [20] discovered that students who reported higher levels of university attachment and satisfaction tended to report better social connections, more social acceptance, and higher levels of academic competence. Likewise, the higher levels and better quality of social support that students received from friends and others, the lower the levels of stress they perceived during the college adjustment processes and the better the predicted adjustment outcomes [43, 44]. There is also strong evidence that social support networks can help individuals adjust to stressors and facilitate coping in the long term, even when facing extremely stressful situations such as the COVID-19 pandemic [45]. As repeatedly shown in the past literature and again highlighted in the results of this study, the importance of a solid social support network comprising friends, family, and even faculty members in mitigating stressors speaks to the necessity of universities allocating more consideration and resources to facilitate students’ social connections as a coping strategy in general and especially during a public health emergency like COVID-19 that emphasizes social distancing.

On the other hand, the results of this study showed that the more students used confrontive and escape avoidance coping strategies, the less likely the students were to feel supported socially during the initial stage of the pandemic. Chao [46] also indicated that people who engaged more in dysfunctional coping perceived more levels of stress, lower levels of social support, and lower levels of well-being. Wishful thinking and social withdrawal also were found to be negatively related to a person’s subjective well-being when encountering stress [47]. Within the context of COVID-19 pandemic adjustment, it is possible that students who reported using more confrontive and escape avoidance coping strategies may have held more negative views towards their situations and social supports. In return, such negative views may have led to a distorted image of the social support required to adapt to the COVID-19 pandemic and a lower level of satisfaction and well-being during that adaptation.

### General COVID-19 regulations response

With regard to students’ response to general COVID-19 regulations imposed by public health officials, the results indicated that the more attached students were to their institutions and the better they had adjusted to their college social lives, the more likely they were to respond positively to public policies and regulations regarding COVID-19 pandemic restrictions. Conversely, the more they used distancing and confrontive coping strategies, the less likely they were to respond positively to regulations. As discussed in past research, students who report higher levels of university attachment and satisfaction tend to possess more coping strategies and less negative psychological distress in general [20], but higher engagement in dysfunctional coping leads to lower levels of well-being [46]. Moreover, Rentner and Alsulaiman [48] discovered that many students held optimistic biases and believed that they were less vulnerable to the virus than their family and friends. The findings of this study again highlight the importance of universities establishing solid institutional and social support as well as providing more positive coping strategies to help their students navigate through the pandemic and adhere to regulatory guidelines.

### Discriminatory impact related to the COVID-19 pandemic

Discriminatory events have occurred repeatedly and been widely scrutinized during the COVID-19 pandemic. The results of this study showed that when students encountered discriminatory incidents stemming from the COVID-19 pandemic, the more attached to their university and the more socially adjusted they were, the more likely they were to perceive a discriminatory impact. This finding in fact contradicts the existing research. Research has revealed that support from faculty, one form of social support, protected undergraduate students from severe depression during COVID-19 [49], and those who were able to virtually connect with others socially were better able to cope with the stress induced by encountering COVID-19-related discrimination [50]. One possible explanation for this contradiction could be that this study’s data were collected during the initial peak of the pandemic when institutional support and virtual social connections had not yet been firmly established by the students. It is also possible that students who relied more on their attachment to their universities and social connections may have exhibited lower levels of self-efficacy [51], which could have played a role in their coping strategies.

This study also discovered that students who used more distancing coping strategies and who were more adjusted in their personal psychological and physical well-being tended to perceive less impact from COVID-19-related discriminatory incidents. This could be due to the disengagement techniques that the students were using during the initial peak of the pandemic. Distancing oneself from an immediate stressor may create the temporary impression of removing the stressor, and yet the stress likely remains and creates an exacerbated impact in the long run [46]. On the contrary, students with better personal-emotional adjustment may practice more action-based positive coping strategies, which could contribute to their resilience against the impact of such incidents. Taken together, these results again show the low efficacy of distancing coping strategies and call for the need for universities to introduce more action-based positive coping strategies to better support their students.

### Limitations and future work

One limitation of our study is that we employed standard, well-established machine learning models and investigated the five psychological domains independently. However, a student’s responses in these five domains could be interconnected. For instance, a student’s emotional state could affect their academic adjustment. Thus, a valuable future undertaking could be to apply more advanced machine learning techniques to exploit the interrelationship among the predictive tasks. To this end, multi-task learning [52] offers a promising solution.

Participants were recruited through convenience sampling by utilizing the researchers’ social networks and connections within higher education. Thus, the sample does not fully represent U.S. census demographics in terms of race, ethnicity, and geographical features, although we made efforts to ensure a diverse participant pool. Additionally, the current study pulled participants from a larger-scale study examining the experiences of university students during the early months of the COVID-19 pandemic (March to June of 2020). Thus, the data might not reflect university students’ experiences during later stages of the pandemic. Future researchers might consider examining university students’ adaptation during different stages of the pandemic. The SACQ measure was originally validated with primarily undergraduate students. Future research should consider the suitability and appropriateness of the SACQ among graduate students.
